# Spark Plasma Sintering of AlN/Al Functionally Graded Materials

**DOI:** 10.3390/ma14174893

**Published:** 2021-08-27

**Authors:** Ziyang Xiu, Boyu Ju, Saiyue Liu, Yiwei Song, Jindan Du, Zhimin Li, Chang Zhou, Wenshu Yang, Gaohui Wu

**Affiliations:** 1 School of Materials Science and Engineering, Harbin Institute of Technology, Harbin 150001, China; xiuzy@hit.edu.cn (Z.X.); 15046054961@163.com (B.J.); wugh@hit.edu.cn (G.W.); 2 State Key Laboratory of Advanced Welding and Joining, Harbin Institute of Technology, Harbin 150001, China; 3 Space Environment Simulation Research Infrastructure, Harbin Institute of Technology, Harbin 150001, China; saiyueliu@hit.edu.cn; 4 Shanghai Research Institute of Radio Equipment, Shanghai 200090, China; juzidemengm@163.com (Y.S.); hit_wangpingping@163.com (J.D.); 15900675393@163.com (Z.L.)

**Keywords:** AlN, spark plasma sintering, aluminium matrix composites, microstructure

## Abstract

In this paper, six-layer AlN/Al gradient composites were prepared by a spark plasma sintering process to study the influences of sintering temperature and holding time on the microstructure and mechanical properties. The well-bonded interface enables the composite to exhibit excellent thermal and mechanical properties. The hardness and thermal expansion properties of the composite exhibit a gradient property. The hardness increased with the volume fraction of AlN while the CTE decreased as the volume fraction of AlN. The thermal expansion reaches the lowest value of 13–14 ppm/K, and the hardness reaches the maximum value of 1.25 GPa, when the target volume fraction of AlN is 45%. The simulation results show that this gradient material can effectively reduce the thermal stress caused by the mismatch of the thermal expansion coefficient as a transmitter and receiver (T/R) module. This paper attempts to provide experimental support for the preparation of gradient Al matrix composites.

## 1. Introduction

Functionally graded materials are composites, for which the volume fraction of the reinforcement constituent changes, and the final composites exhibit gradually changing microstructure and properties [1,2,3,4]. The functionally graded materials are different from any of the individual parts that form them, and they exhibit unique properties.

Aluminium alloys are widely used in transport, architecture, and electronic packaging fields due to their low density, good mechanical properties, and high thermal conductivity. In the electronic packaging field, the case of the transmitter and receiver (T/R) component material is generally made of 6061 Al alloy. However, the high thermal expansion coefficient (CTE) of 6061 Al does not match the CTE of the ceramic substrate and will cause a large amount of thermal stress. One effective way to control the CTE of aluminium is by adding low CTE ceramic phases, such as SiC [5,6] and AlN [7], into the Al matrix. These functionally graded AlN/Al composites have been widely studied, as they can achieve the gradual distribution of the volume fraction and CTE. Compared with traditional uniform composites, the composition and organization of functionally gradient composites are continuously gradient distribution, and their performance changes continuously along the gradient direction, which has important application value [8,9]. One side contains high volume fraction of AlN, and a low CTE was connected with the low thermal expansion of low temperature co-fired ceramic (LTCC) inside the T/R components. The other side, with lower volume fraction of the AlN/Al composite, is sealed with the T/R case (6061 Al). Introducing this gradient structure in the electrical packaging field can be expected to reduce the giant thermal mismatch stress and extend service life [10].

Squeeze casting, hot press sintering, and spark plasma sintering (SPS) are the main manufacturing methods for ceramic reinforced Al matrix composites [11,12,13,14,15]. SPS is a novel sintering method that can achieve rapid temperature changes and requires short holding times to densify the material [15]. Ehsan Ghasali et al. reported that the Al-SiC-TiC prepared by SPS shows homogeneous distribution without obvious voids and cracks compared with the conventional sintering method [16]. Other ceramic reinforced Al matrices fabricated by SPS show better mechanical and thermal properties [17,18]. Moreover, it has been reported that a temperature gradient field is formed in the gradient composite by SPS, which is beneficial to forming functionally graded materials [2]. It is necessary to optimize the process parameters of SPS as the sintering processes required for the different volume fractions of AlN reinforced composites are quite different. Sintering temperature and holding time are the two most important sintering parameters. Higher sintering temperature can provide greater driving forces for the densification of AlN/Al composites, and the increase in holding time can reduce the number and size of pores, which is conducive to improving the mechanical and thermal properties of composites [19,20].

In this study, the influences of sintering temperature and holding time on the microstructure of AlN/Al composites were investigated. The six-layered gradient materials were successfully sintered using the SPS process. The microstructure and thermal and mechanical properties of the gradient composites were characterized.

## 2. Materials and Methods

### 2.1. Materials

**Figure 1:**
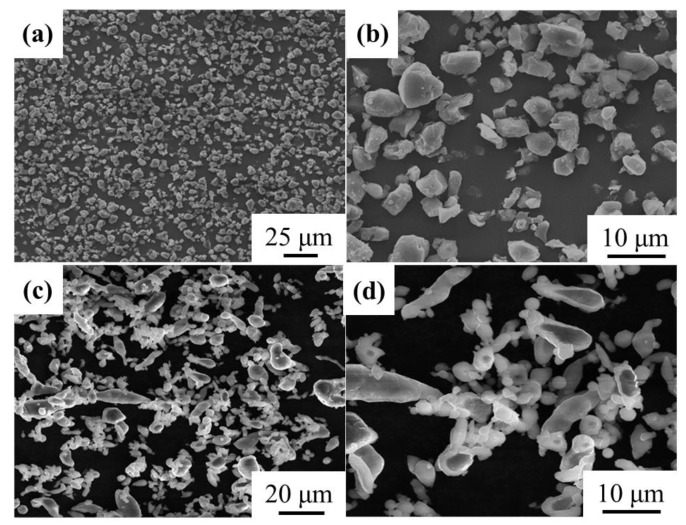
The morphology of AlN and Al powders. (**a**) Low and (**b**) high magnification of AlN powders; (**c**) Low and (**d**) high magnification of Al powders.

AlN particles (99.9%, Yinuo New Material Co., Ltd. Jiangxi, China) and gas-atomized Al powders (Northeast Light Alloy Co., Ltd. Harbin, China) were used as the reinforcement and matrix, respectively. As shown in Figure 1, AlN powders exhibited near spherical morphology with an average diameter of 8 μm, and Al powders had long rod shapes with an average diameter of 10 μm.

**Figure 2:**
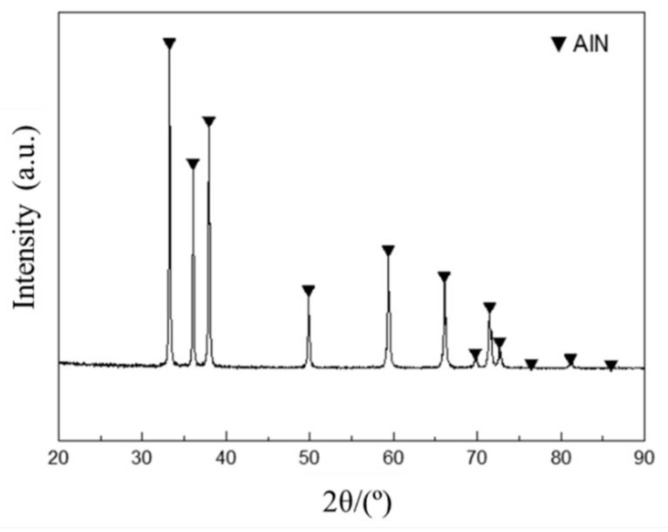
XRD pattern of AlN powder.

XRD patterns of the composites were observed on a Philips X’Pert-PRO using Cu Ka radiation in the range of 20–90°. The CTE of samples were investigated on a NETZSCH Dilatometer 402 °C at a heating rate of 5 °C/min from 20 to 300 °C. The dimensions of each specimen are those of a φ6 mm × 25 mm cylinder. Microstructures were studied by scanning electron microscopy (SEM FEI Helios 600 i). The relative density was measured by the Archimedes method. The Vickers hardness measurement was conducted on the Laizhou HBV-30 A type Vickers machine with a 250 N load for 30 s. All of the tests were performed on at least five samples to improve the statistical significance of the results. The XRD results (Figure 2) show the main peaks of AlN, and almost no impurity peaks can be seen. The quantitative analysis results show that the AlN powders only contain 1.2 wt.% oxygen, and other impurity elements are present at less than 50 ppm.

### 2.2. Fabrication Process

**Figure 3:**
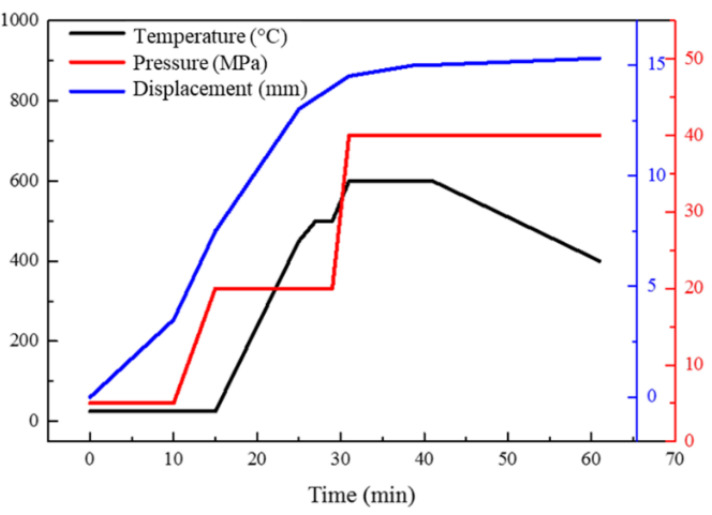
Actual temperature-time and displacement-time curves of the AlN/Al composites.

The six-layered gradient composites were fabricated by ball milling and the SPS process. The process of SPS is to load the raw powder into the mould, and use the pulse current to perform in situ discharge sintering at the contact interface of the reinforcement and the matrix. Compared with the traditional preparation technology, SPS technology has the unique advantages of low preparation temperature, in situ sintering of the interface, and controllable interfacial reaction. The mixed AlN and Al powders were processed at a rotating speed of 150 rpm for 2.5 h with ball-to-powder weight ratio of 5:1. Then, the mixed AlN/Al powders were moved into a graphite mould under 5 MPa pressure. The preform was sintered in the equipment of FCT Systeme GmbH. The on-off ratio was 2:1. Temperatures of 550 °C, 575 °C, 600 °C, and 625 °C were selected as target temperatures to study the effect of sintering temperature on properties. During the heating stage, the pressure first reached 10 MPa, and then quickly increased to 20 MPa. The final pressure was 40 MPa when the sintering temperature reached the target value. The infrared thermometer was used to detect the upper head’s temperature. The specific temperature/displacement curve is shown in Figure 3. The pressure displacement and moving rate of the indenter are generally positively correlated with the sintering temperature, holding time, and applied pressure in the pre-sintering stage.

## 3. Results and Discussion

**Figure 4:**
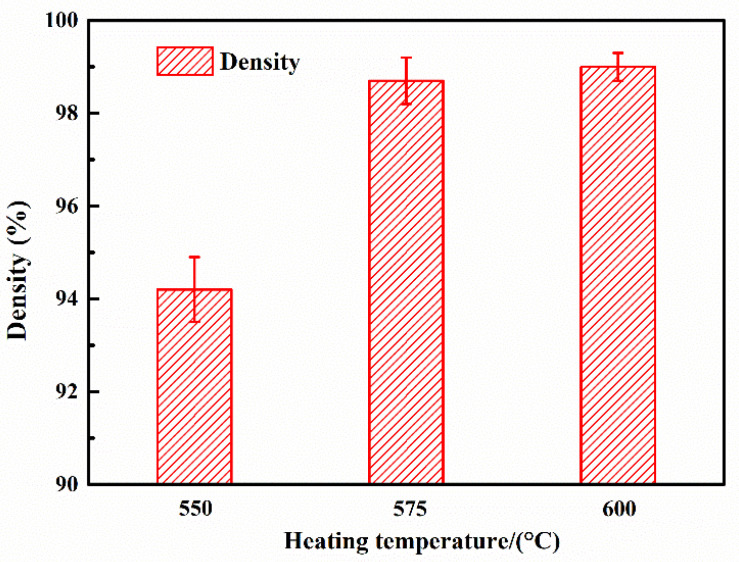
The relationship between relative density and fabrication temperature in the 25 vol.% AlN/Al composite.

In order to control the properties of gradient AlN/Al composites, the composite preparation process was studied. First, the sintering temperature was fully investigated. The effect of the sintering temperature on the microstructure of 25 vol.% AlN/Al composites was studied. As the melting point of pure Al is 660 °C, four different sintering temperatures of 550 °C, 575 °C, 600 °C, and 625 °C were used to investigate the 25 vol.% AlN/Al composites. The relationship between temperature and relative density is shown in Figure 4.

As seen from Figure 4, as the sintering temperature increased from 550 °C to 575 °C, the relative density of 25 vol.% AlN /Al composite increased from 94.2% to 98.7%, further reaching 99% as the temperature further increased to 600 °C. However, when the sintering temperature reached 625 °C, an abnormal movement of the upper pressure head occurred, and the furnace cavity was opened with a large amount of aluminium extrusion. This is as the sintering temperature reached 625 °C, the Al matrix has begun to melt. Under the action of pressure, it was extruded from the gap of the die, and the sintering of the composite was no longer possible at this time. Therefore, the most suitable sintering temperature in this paper is 600 °C.

Second, the relationship between holding time and relative density was investigated. It is well known that, among the gradient materials, high volume fractions of the AlN/Al layer are more difficult to sinter. Hence, it is instructive to investigate the relationship between the relative density of high-volume fractions of the AlN/Al composites and SPS holding time. Based on previous research, the most suitable sintering temperature is 600 °C. The six different holding times of 10 min, 15 min, 20 min, 25 min, 30 min, and 35 min were used to investigate the most suitable sintering parameters.

**Figure 5:**
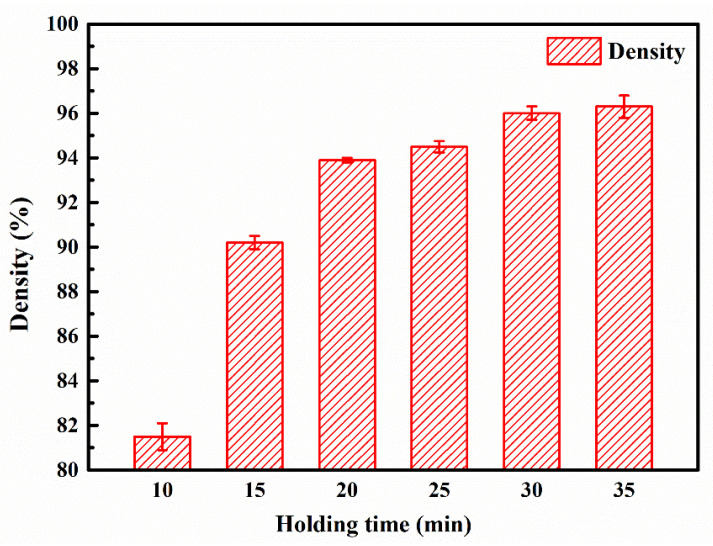
The relationship between relative density and holding time in the 45 vol.% AlN/Al.

**Figure 6:**
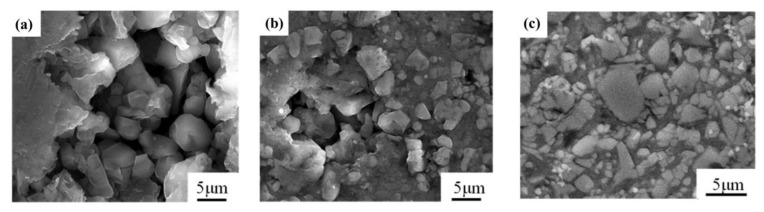
SEM figure of the 45 vol.% AlN/Al composites with different holding times: (**a**) 10 min; (**b**) 20 min; and (**c**) 30 min.

As shown in Figure 5, the relative density increased continuously with the increase in holding time. When the holding time was extended from 10 min to 30 min, the relative density increased from 81.5% to 96.1%. The SEM results (Figure 6) show the same trend, namely, that the pores decreased as the holding time gradually increased, and almost no pores could be observed in the case of 30 min holding time. It can also be seen from Figure 6 that the AlN particles were distributed evenly in the Al matrix without agglomeration. However, the density only slightly increased to 97% when the holding time was extended to 35 min. This phenomenon has also been reported in the sintering process of other composites. Wang and Gürbüz et al. [21,22] observed in B_4_C/Al and Graphene/Al composites that in an appropriate temperature range, increasing the sintering temperature and prolonging the sintering time, respectively, will significantly increase the density of the composite, thereby improving the performance. However, excessively increasing the sintering temperature leads to a significant decrease in material properties. The grain growth and interface reaction will become stronger with the increase in the holding time, which may weaken the mechanical properties. Therefore, the most suitable preparation time for this paper is 30 min.

**Figure 7:**
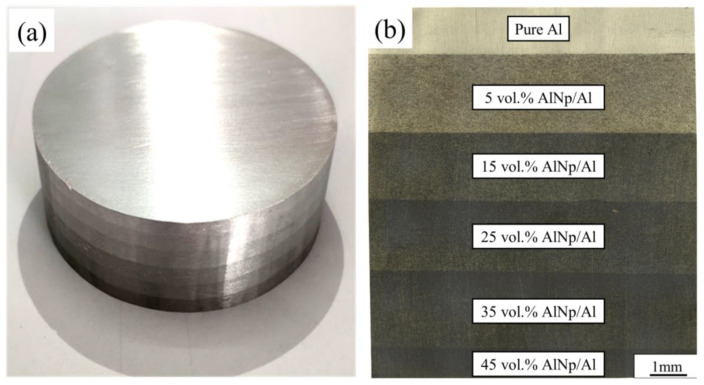
(**a**) Sintered gradient AlN/Al composites with a size of φ64 mm × 18 mm; (**b**) metallography image of a six-layer gradient composite.

**Figure 8:**
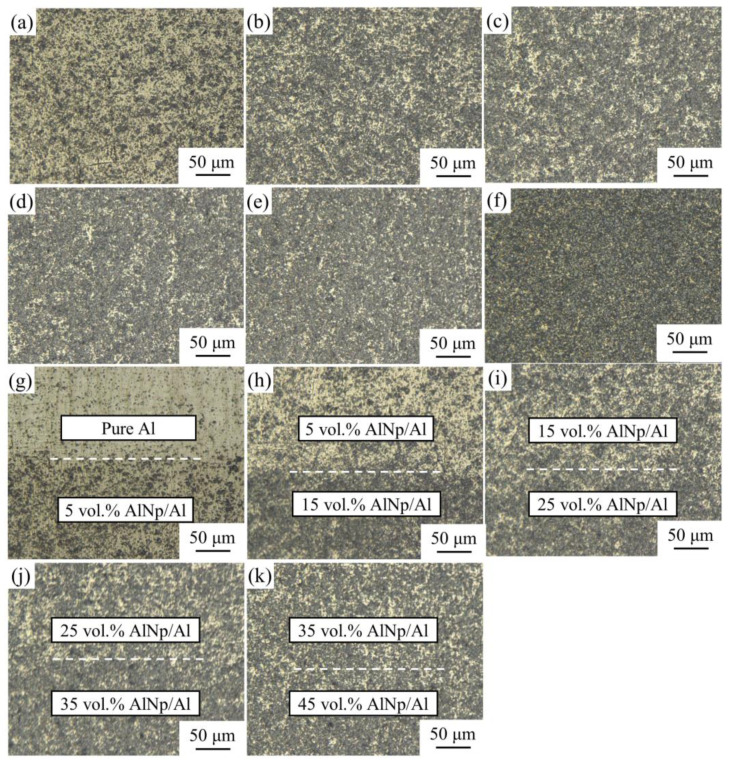
SEM imaging of gradient composites with different volume fraction of AlN: (**a**) 5 vol.% AlN/Al; (**b**) 15 vol.% AlN/Al; (**c**) 25 vol.% AlN/Al; (**d**) 35 vol.% AlN/Al; (**e**) 45 vol.% AlN/Al; and (**f**) 55 vol.% AlN/Al; SEM photomicrographs of layer regions: (**g**) Pure Al–5 vol.% AlN/Al; (**h**) 5–15 vol.% AlN/Al; (**i**) 15–25 vol.% AlN/Al; (**j**) 25–35 vol.% AlN/Al; and (**k**) 35–45 vol.% AlN/Al.

Based on the above experimental results, the selected SPS sintering parameters (sintering temperature of 600 °C, holding time of 30 min) were used to sinter the gradient AlN/Al composite. Metallographic and SEM observations were performed on six layers AlN/Al layered gradient composites, as shown in Figure 7 and Figure 8.

Figure 7b is an enlarged photo of the interface metallography of a six-layer gradient composite. From top to bottom are the pure Al, 5 vol.% AlN/Al, 15 vol.% AlN/Al, 25 vol.% AlN/Al, 35 vol.% AlN/Al, and 45 vol.% AlN/Al. The layers with higher volume fractions of AlN are darker in the picture. It can be seen from Figure 8 that no interfacial cracks or holes exist between the layers, and the interface line is quite smooth and straight. No holes or cracks can be seen around the interface. These images indicate that the AlN particles in each layer are evenly distributed without agglomeration. The combination of non-porous interfaces is beneficial to ensure that the performance of the composite remains continuous in the gradient direction, and the AlN/Al gradient composite can better play the characteristics of its gradual change in performance [2]. Moreover, it can be seen from Figure 8 that no obvious holes can be seen in the 5–35 vol.% AlN/Al composites, as indicated by the dense structure and the relative density results.

**Figure 9:**
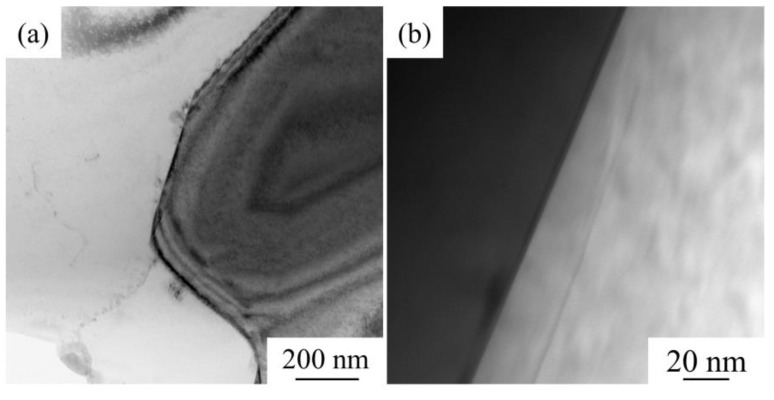
TEM images of sintered 25 vol.% AlN/Al composite: (**a**) low magnification image, where the Al matrix is white and AlN particles are black; (**b**) high magnification image, where the Al matrix is black and AlN particles are white.

Furthermore, the interface between the reinforcement and aluminium matrix is crucial in the composite, as a well-bonded interface can transfer stress and guarantee excellent mechanical properties. Figure 9 shows the TEM results of the interface of 25 vol.% AlN/Al composite. TEM observations show that the interface between AlN and Al is clean and tightly bonded without voids or interfacial cracks. As shown in Figure 9a, some dislocations appeared in the Al matrix, which may strengthen the composite during plastic deformation. The interface of the composite material obtained in this study is a direct bonding interface of AlN-Al, and there is no reaction phase at the interface. Similar phenomena have also been observed by other researchers. Wang et al. [23] studied the AlN-Al interface prepared by magnetron sputtering, and also observed clean AlN-Al interface bonding. Han [24] and Bian [25] et al. found that when multi-reinforcement synergistically reinforces Al matrix composites, the AlN-Al interface is also dominated by direct bonding interfaces without interfacial reactions. However, Yang et al. [26,27] found that in Zr-containing alloy systems, AlN easily reacts with Zr to form Al_2_Zr and ZrN. In the choice of the base alloy, this study chooses a Zr-free system to protect AlN from reacting with the base alloy and to preserve the clean interface bonding of AlN-Al to the greatest extent.

Good interfacial bonding in the composite ensures strong plasticity and excellent thermal properties. In particles, poor interface bonding can lead to micro-cracks and premature failure in the electronic packaging field. In this work, the mechanical and thermal properties of gradient composite were characterized by their hardness and thermal expansion behaviours.

**Figure 10:**
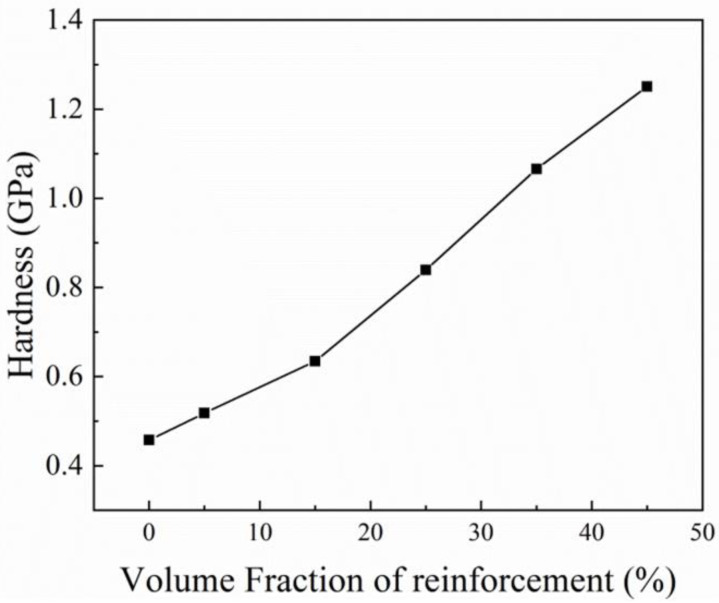
Hardness of AlN/Al gradient composite with different volume fractions.

The Vickers hardness as a function of AlN volume fraction is shown in Figure 10. It can be found that the hardness increases gradually with the increasing amount of AlN. The hardness of pure Al is only 0.46 GPa, but it increases dramatically to 1.25 GPa upon the addition of 45 vol.% AlN, namely, by a nearly three-fold increase. The hardness data show that the performance of the graded material exhibits a gradient change after an optimized process.

**Figure 11:**
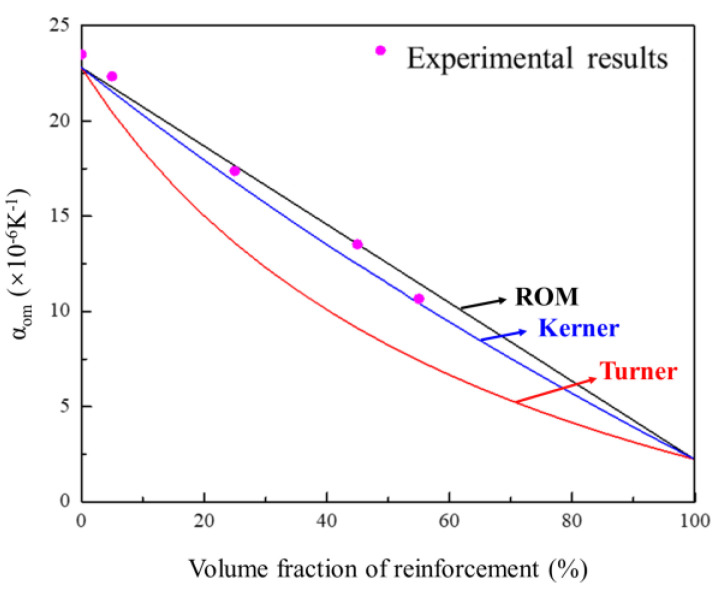
Calculated and measured CTE values of AlN/Al gradient composites with different volume fractions.

At the same time, the addition of a high content of ceramic reinforcing phase can effectively control thermal expansion of Al matrix. The experimental thermal expansion results are shown in Figure 11. It can be found that the CTE decreases linearly with the increasing volume fraction of the AlN. Rule of mixture (ROM) [28], Turner [29], and Kerner [30] models are the most widely used CTE models to predict the thermal expansion value [31]. Figure 11 indicates that the experimental CTE results were in good agreement with the theoretical values. The ROM provides the upper bound, and the Turner rule provides the lower bound. In particular, as the AlN content increased to 45 vol.%, the CTE of composite was only 13–14 ppm/K, which was only half that of the pure Al (23 ppm/K) matrix.

In the T/R components, the cover was made of 6061 Al, which has a high CTE value of 22–24 ppm/K, but the LTCC consisted of low CTE ceramics (CTE <10 ppm/K). The giant thermal expansion mismatch between 6061 Al and LTCC will lead to large thermal stress during thermal cycles in practical use. Assuming that this component is undergoing a (–100)–100 °C thermal cycle, the accumulated residual stress can be calculated by using the following equation: (α_Al_ − α_LTCC_) × ΔT × E, where α_Al_ and α_LTCC_ are the thermal expansion coefficients of Al and LTCC, respectively, T is the temperature, and E is the modulus of elasticity. The maximum calculated value can reach ~150 MPa, which will greatly reduce the life of the device.

**Figure 12:**
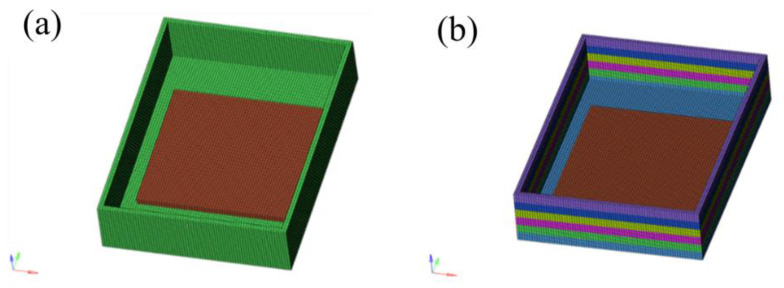
The simulated package cover in a T/R component: (**a**) 6061 Al; (**b**) gradient AlN/Al composite; different colours refer to different AlN/Al composites.

The gradient composite which has a gradient CTE can effectively reduce thermal stress. To investigate the influence of the gradient composite on the reduction in thermal stress, the MSC. Marc software was used to simulate the thermal stress values of the traditional 6061 Al cover and gradient AlN/Al cover applied to the T/R package. As shown in Figure 12, the T/R models are as follows: the package cover size is 52.5 mm × 37.5 mm × 12 mm with a thickness of 1 mm; the AlN ceramic substrate, which was placed on the bottom surface, is a 30 mm × 30 mm × 1.5 mm plate. A 13 mm × 13 mm cuboid heat source (150 W) is loaded on the AlN ceramic substrate.

**Figure 13:**
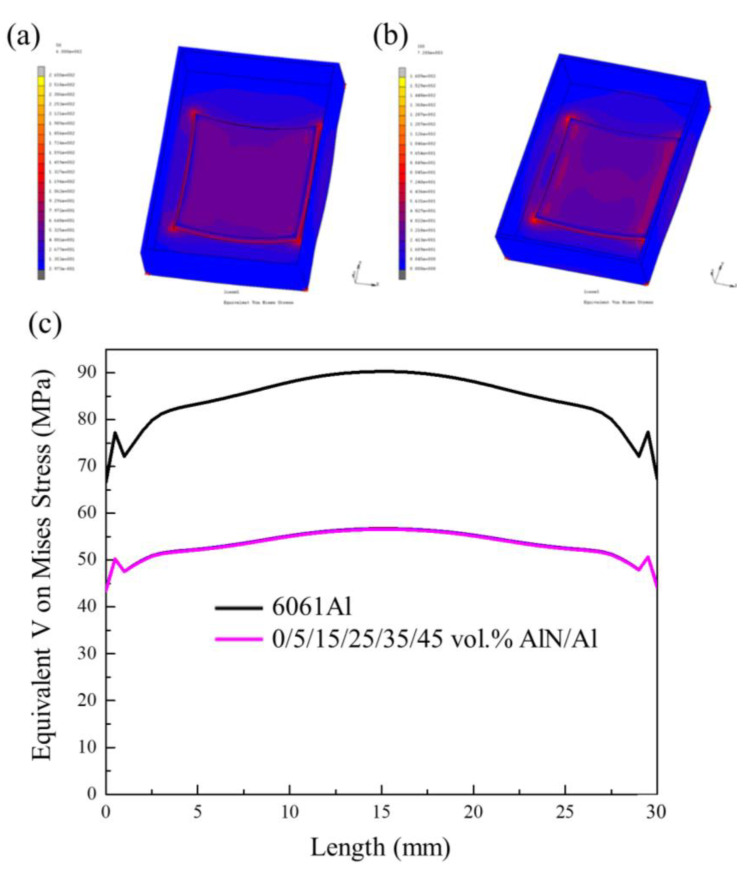
(**a**,**b**) von Mises stress mapping; (**c**) values of 6061Al and gradient composites.

The thermal stress values in this paper were characterized by the von Mises stress. Von Mises stress is an equivalent stress based on shear strain energy, which is used to describe changes in the stress distribution in the model [32]. The specific value of the von Mises stress was taken as a line across the AlN substrate. As shown in Figure 13, the simulated mean von Mises stress was 90.3 MPa when using the 6061 Al as the cover. The von Mises stress of the gradient AlN/Al composite is only 56.6 MPa, which is approximately 40% lower than that of the 6061 Al system. The simulation values confirm that the gradient material, which has a gradient thermal expansion property, can effectively reduce the thermal mismatch stress.

## 4. Conclusions

Currently, investigation on the functionally graded Al matrix composites with high reinforcement content variation (more than 30 vol.% difference) prepared by SPS method has been rarely reported, as the optimized process parameters for each layer with different volume fractions would be different. In the present work, the preparation of gradient AlN/Al composite by SPS method was explored for the first time. The sintering temperature for six-layer AlN/Al gradient composites with AlN content varies from 0 to 45 vol.% has been optimized to be 600 °C, and dense gradient AlN/Al composite was obtained by one-step sintering using the optimized process. Moreover, the hardness and thermal expansion also showed gradient properties with cross composites. The simulation results indicate that this gradient material can effectively reduce the thermal stress caused by the mismatch of the thermal expansion coefficient, which is very attractive for the application in transmitter and receiver (T/R) module.

Therefore, the present work demonstrates that it is feasible to prepare Al matrix composites with high reinforcement content variation (higher than 45 vol.% difference) by one-step SPS method, which provides a new method for the preparation of functionally graded composites. Moreover, the prepared gradient AlN/Al composite would also be used in thermal management field, which requires a lower coefficient of thermal expansion to match the semiconductor, and a higher coefficient of thermal expansion to match the installation platform.

## Data Availability

The data presented in this study are available on request from the corresponding author.
